# Konjac Glucomannan from *Amorphophallus konjac* enhances immunocompetence of the cyclophosphamide‐induced immunosuppressed mice

**DOI:** 10.1002/fsn3.2038

**Published:** 2020-12-04

**Authors:** Jiajia Dai, Jian Chen, Jun Qi, Mengru Ding, Wei Liu, Taili Shao, Jun Han, Guodong Wang

**Affiliations:** ^1^ School of Public Health Wannan Medical College Wuhu China; ^2^ Anhui Provincial Engineering Research Center for Polysaccharide Drugs, Provincial Engineering Laboratory for Screening and Re‐evaluation of Active Compounds of Herbal Medicines in Southern Anhui, Anhui Province Key Laboratory of Active Biological Macromolecules Wuhu China; ^3^ College of Tea & Food Science and Technology Anhui Engineering Laboratory for Agro‐products Processing Anhui Agricultural University Hefei China; ^4^ School of Pharmacy, Drug Research & Development Center Wannan Medical College Wuhu China

**Keywords:** *Amorphophallus konjac*, cyclophosphamide, immunocompetence, immunosuppressed, Konjac glucomannan

## Abstract

This present study was designed to evaluate the immunomodulatory activity of Konjac glucomannan (KGM) on immunosuppressed mice induced by cyclophosphamide (CTX) treatment. The mice immunodeficiency model was established by CTX. KGM was used to modulate the activities of immunosuppressive mice. It was proved that KGM could promote the proliferation of lymphocyte, thymus, and spleen indices, and alleviate the atrophy of immune organs and weight loss. Besides, in mice serum, the levels of cytokines including TNF‐α, IgG, IL‐2, and the contents of hemolysin were also increased after treatment with KGM. Furtherly, in nonspecific immunity, KGM could enhance natural killer (NK) cell lethality and pinocytic activity of mouse peritoneal macrophages. Therefore, all of these results revealed that KGM could improve the reduced immunity of CTX‐induced mice via modulation innate immunity and adaptive immunity.

## Graphical abstract

This present study was designed to evaluate the immunomodulatory activity of Konjac glucomannan (KGM) on immunosuppressed mice induced by cyclophosphamide (CTX) treatment. It was found that KGM could improve the reduced immunocompetence of CTX‐induced mice via modulation innate immunity and adaptive immunity.

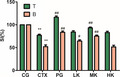

## 1 INTRODUCTION

The immune system which is a physiological function is consisted of immune organs, immune cells, and immune molecules, including nonspecific, and specific immunity according to the source of the immunization (Li et al., 2017). The immune response in human, as a natural defense barrier, could clear away aging, damage, death, and mutated cells. Foreign matters are identified and treated by physiological response identifying and eliminating “alien” (Zhou et al., 2018). In different pathophysiological conditions, immunosuppression of the bodies may be observed, when it is difficult that the immune system protects the bodies from threatening.

Cyclophosphamide (CTX) causing bone marrow suppression, immunosuppression, oxidative stress, and other side effects, as an alkylating anticancer agent, is extensively used in the treatment of various cancers and disorders, such as systemic lupus erythematosus, lymphoma, and autoimmune diseases (Lee et al., 2018; Zhong et al., 2006). CTX administration will damage normal cells, leading to decrease body weight, relative weights of spleen and thymus, leukocyte and natural killer (NK) cell activity, and then inhabit immune organ function, resulting in secondary infections (Ehrke, 2003). Besides, it also caused the imbalance of various blood cells in the peripheral blood. All of these eventually will lead to immunosuppression. The host immune response depends on the above important immune organs and immune cells called immune defense and plays a vital role in the enhancement of the body's immunity (Bischoff & Krämer, 2007). Therefore, it is necessary that immunomodulatory agents are used to reduce the CTX‐induced immunosuppression.

For years, natural polysaccharides, as biological response modifiers, widely distributed in higher plants, algae, microorganisms, and animals. Because of the various bioactivities and functions, polysaccharides have attracted more and more attention, playing an important role in antioxidant, antitumor, antidiabetic effects, and immunomodulation (Chen et al., 2019). Polysaccharides with low toxicity and negligible side effects are selected as an ideal immunomodulatory agent (Mizutani & Yoshida, 1991). It has been found that polysaccharides can enhance immunity by promoting specific organs and cells by stimulating natural killer cells, T cells, B cells, and macrophage in the immune system (Tan et al., 2016). So, we can see that polysaccharides could be widely used in food and medicine fields.

Konjac glucomannan (KGM), a perennial plant belonging to the family Araceae, was obtained from tubers of Amorphophallus konjac. KGM, as a nutritional supplement, plays a very important role in reducing cholesterol, improving blood sugar levels, and immune function, and so on. It has been demonstrated that KGM could prevent the development of allergic rhinitis‐like symptoms by increasing the content of IgE and IgG levels in mice (Al‐Ghazzewi & Tester, 2010). Several scholars have reported that KGM can inhibit the development of atopic diseases by reducing the production of IgE in mice. Previous studies have reported that KGM modulated immune organ, and provided humoral, cellular, and mucosal immunity. KGM also has played an important in enhancing the process of biological detoxification in animals by significantly influencing albumin, total protein, activity of creatinine, aspirate aminotransferase, and alanine aminotransferase levels (L.‐G. Chen et al., 2005; Onishi et al., 2007). However, it has not been found that KGM could reduce damage and enhance the immunity of immunosuppressed mice induced by CTX treatment. Therefore, this present study furtherly focused on evaluating the modulation activities of KGM from *Amorphophallus konjac* on the CTX‐induced immunosuppressive mice.

## 2 MATERIALS AND METHODS

### 2.1 Materials

Konjac glucomannan was purchased from Sanyuan Tianyu biological products co. LTD (Xian, China). KGM which is a kind of natural neutral polysaccharide is mainly composed of D‐glucose (G) and D‐mannose (M) monomers in M/G molar ratio of 1.5–1.6 connecting by β‐1,4 glycosidic bonds with about 1 acetyl group in every 17–19 sugar units at the C‐6 position. Fetal bovine serum (FBS), Dulbecco's modified Eagle's medium (DMEM), and RPMI‐1640 were purchased from GIBCO BRL (Carlsbad, CA, USA). CTX, 3‐(4, 5‐dimethyl‐thiazol‐2‐yl)‐2, 5‐diphenyl‐tetrazolium bromide (MTT), and levamisole (LMS) were bought from Sigma‐Aldrich (St. Louis, USA). Concanavalin‐A, Mouse TNF‐α ELISA kit, Mouse IL‐2 ELISA kit, and Mouse IgG ELISA kit were provided by Boster Biological Technology (Wuhan, China). YAC‐1 cells, sheep red blood cells (SRBC), neutral red dye, trypan blue, and complement were obtained from Beijing Suolaibao Biological Engineering co. Ltd (Beijing, China). Penicillin, pancreatic enzymes, and Cell Counting Kit‐8 (CCK‐8) were bought from Shanghai Yisheng Biotechnology co., Ltd (Shanghai, China).

### 2.2 Animal

Female‐specific pathogen‐free (SPF) BALB/c mice (6–8 weeks old, 18–22 g) were purchased from Beijing Weitong Lever Laboratory Animal Technology co. Ltd (Certificate No. SCXK (Jing) 2016–0006, Beijing, China). The mice were acclimatized for a week under standard husbandry conditions (temperature: 22 ± 2°C, humidity: 55 ± 5%) in the animal house. The mice were casually fed with food and water during the experiment. Ethics approval was obtained from the ethics committee of Wannan Medical College (approval number 2018‐LLSC‐001).

### 2.3 Experimental design

The immunosuppressive model was established by CTX according to the previous study (Gao et al., 2019). After 7‐day adaptation, the mice were randomly classified into six groups (*n* = 8 per group), including control group (CG), CTX model group (CTX) positive group (PG), and the low, middle, and high doses of KGM. In the first three successive days, the control group was treated by physiological saline once daily, the other groups were successively injected by CTX at 40 mg/kg/day, and then, injected by CTX at 80 mg/kg/day every seven days. The mice were respectively administered orally with KGM at 200 mg/kg/day, 300 mg/kg/day, and 400 mg/kg/day, from the fourth to the twenty‐eighth day. The mice in the positive group were administered 40 mg/kg/day of LMS. After the last drug treatment, the mice were forbidden to eat, but water, within 12 hr.

### 2.4 Determination of thymus and spleen indices

After 28 days, BALB/c mice were weighted and then killed through cervical dislocation. Thymus and spleen were collected, weighted, and calculated the spleen and thymus indices according to the following formula (Gao et al., 2019).

$$\text{Index}\left(\text{mg}\;\text{per}\;10\;g\right)=\frac{\text{weight}\;\text{of}\;\text{thymus}\;\text{or}\;\text{spleen}(\text{mg})}{\text{body}\;\text{weight}(g)}$$

### 2.5 Proliferation of spleen lymphocytes

ConA and LPS, respectively, induced spleen lymphocyte proliferation was used to evaluate T and B lymphocyte proliferative responses. The spleen lymphocytes were obtained according to Wang et al. (2016). In brief, mice were soaked in ethyl (75%) for 5 min, and then, spleen removed was ground and washed by PPS, whose filter liquor was centrifuged at 120 *g*/min for 5 min. The resuspension was cultured in RPMI‐1640 with 10% fetal bovine serum and 1% penicillin and then plated in a 96‐well culture plate with 100 μl/well (6 × 10^6^ cells/ml). Cultured with Con A (10 μl/ml) or LPS (10 μl/ml), and incubated at 37°C and 5% CO_2_ for 72 hr, 10 μl of CCK‐8 solution (5 mg/ml) was added to each well and then continued to hatch for 2h. The optical density (OD) of suspension was measured by microplate reader at 490 nm (Raj & Gothandam, 2015; Wang et al., 2016). The proliferation of spleen lymphocytes was calculated by stimulation index (SI).

$$\text{SI}=\frac{{\text{OD}}_{\text{SW}}}{{\text{OD}}_{\text{CW}}}$$

where OD_SW_ was the optical density of the sample well, OD_CW_ was the optical density of the control well.

### 2.6 Level of serum hemolysin

On the twenty‐fourth day, the mice from were immunized by injecting a suspension of sheep erythrocytes (SRBC) (2%, 0.2 ml). After four days, the mice blood in eyes were collected at 4°C for 12 hr, and serum separated dilute with 200 times saline. 1 ml of 10% SRBC and 10% complement 1 ml were added to the dilute mentioned above to centrifuge tube in a water bath at 37°C for 30 min. The dilute was centrifuged at 3,500 *g* for 10 min (Sevko et al., 2013). The absorbance of suspension collected was measured by ultraviolet spectrophotometer at 540 nm.

### 2.7 Analysis of the NK cell lethality

The lethality of NK cell was determined according to previous reported (Wang et al., 2016). NK‐target YAC‐1 mice lymphoma cells were cultured and adjusted to 1 × 10^5^ cells/well by RPMI‐1640. Splenocytes, as effector cells, were added in 96‐well plates at the density of 5 × 10^6^ cells/well at a ratio of 50:1. After the cells incubated at 37°C for 4 hr, every well was added in 10 μl/ml CCK‐8 and then continued to hatch for 2 hr (Wang et al., 2017). The optical density (OD) of suspension was measured by microplate reader at 490 nm (Li et al., 2017).

The NK cell kill rate (KR) was calculated as follows:

$$\text{KR}=\left(1‐\frac{{\text{OD}}_{S}‐{\text{OD}}_{E}}{{\text{OD}}_{T}}\right)\times 100\%$$

where OD_T_ was the optical density of the control target cells, OD_S_ was the optical density of test samples, and OD_E_ was the optical density of the control effector cells.

### 2.8 Pinocytic activity of mouse peritoneal macrophages

The phagocytic capacity of macrophages was determined by neutral red test (Sevko et al., 2013; Zhang et al., 2020). Cells were cultured for at 37°C in 5% CO_2_ for 24 hr, and 100 μl neutral red (0.1%) was added into each well for 1 hr. The neutral red was washed with PBS for 3 times. 100 μl cell lysate (50% ethanol: 1% acetic) was put into each well, stored for 3 hr. The optical density (OD) of suspension was measured by microplate reader at 559 nm.

The phagocytic index (PI) was calculated as follows:

$$\text{PI}=\frac{{\text{OD}}_{\text{SG}}}{{\text{OD}}_{\text{CG}}}\times 100\%$$

where OD_SG_ was the optical density of the sample group, and OD_CG_ was the optical density of the control group.

### 2.9 Determination of cytokines

The concentrations of TNF‐α, IgG, and IL‐2 were measured by ELISA kit in previous reported. BALB/c mice in each group were killed by cervical dislocation, and then, eyeball blood was collected. The blood samples were centrifuged at 3,000 *g* for 10 min to draw the upper serum (Bujalance et al., 2007). The contents of cytokines TNF‐α, IgG, and IL‐2 were measured by ELISA kit according to the instructions.

### 2.10 Statistical analysis

All of the data were carried out at least three independent experiments. Values were presented as mean ± *SD*. All statistical results were made by one‐way analysis of variance (ANOVA) and Dunnett's test. The *p* of value less more than .05 presented statistically significant.

## 3 RESULTS AND DISCUSSION

### 3.1 Spleen and thymus indices

Thymus is the main central immune organ of the organism, which plays an important regulatory role in peripheral immune organs and immune cells. The spleen, as the largest peripheral immune organ, immunomodulates on the organism by the phagocytic action of macrophages, T‐cell‐mediated cellular immunity, and B‐cell‐mediated humoral immunity (Wang et al., 2013). T lymphocytes were mainly differentiated, developed, and matured in the thymus, but all of the above T and B lymphocytes were accomplished in the spleen. The indexes of them could reflect immune function to some degree (Wang, et al., 2012). The spleen and thymus indices were as shown in Table 1. CTX group was much lower than control group, which represented the worse immune activity. Compared with CTX group, KGM could improve the immune activity. In all the intervention groups, the middle dose of KGM treatments extremely significantly enhanced the spleen indices (*p* < .01) and significantly enhanced the thymus indices (*p* < .05), obviously closing to control group. As we known as, the immune organ indices which are original indicators could reflect immune capabilities. In this study, we can see that KGM could significantly alleviate the becoming small the spleen and thymus indices, and weight loss. These results demonstrated that KGM could repair and reverse the CTX treatment immunodeficient mice.

**Table 1:** Effects of KGM on body weight and thymus and spleen indices in CTX‐induced immunosuppressive mice

Group	Initial weight (g)	Final weight (g)	Thymus index (mg/g)	Spleen index (mg/g)
CG	19.32 ± 0.39	25.48 ± 0.47	2.06 ± 0.20^a^	2.55 ± 0.40^b^
CTX	19.47 ± 0.37	20.9 ± 0.60	1.50 ± 0.51^c^	1.84 ± 0.21^c^
PG	19.05 ± 0.53	23.02 ± 1.30	2.01 ± 0.15^a^	2.37 ± 0.39^b^
LK	19.23 ± 0.33	21.30 ± 0.48	1.87 ± 0.18^ab^	1.95 ± 0.26^c^
MK	19.03 ± 0.55	22.33 ± 0.87	2.01 ± 0.21^a^	3.12 ± 1.20^a^
HK	19.39 ± 0.38	23.02 ± 0.77	1.82 ± 0.17^ab^	2.17 ± 0.30^b^

Note

Values are presented as means ± *SD*. Values in the same row with a different superscript differ significantly at *p* < .05.

Abbreviations: CG, control group; CTX, cyclophosphamide; HK, High ‐ KGM dose group with 400 mg; LK, Low ‐ KGM dose group with 200 mg; MK, Middle ‐ KGM dose group with 300 mg; PG, positive group.

### 3.2 Effect on splenic lymphocyte proliferation

**Figure 1:**
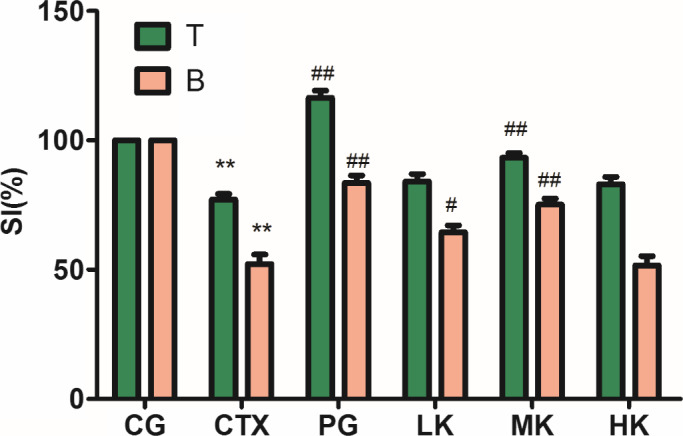
KGM demonstrated significant influences on splenic lymphocyte proliferation including T and B cells induced by Con A or LPS. Values are expressed as means ± *SD*, *n* = 8. *p < .05, **p < .01, *compared with control group; ^#^ *p* < .05; ^##^ *p* < .01, Compared with CTX group. CG, control group; CTX, CTX model group; PG, positive group; LK, low KGM; MK, middle KGM; HK, high KGM

T and B lymphocyte, which could be used to evaluate the degree of immunity of mice, plays a very important role in humoral immunity and cell immunity (Wang, Meng, et al., 2012). To investigate the specific immune function of lymphocytes, the proliferation of T and B lymphocyte was induced by Con A and LPS, respectively, to enlarge, divide, and reproduce, and perform the functions of cellular immunity and humoral immunity (Mathieu et al., 2016). T lymphocyte, as cellular immunity, could reject and transplant the foreign tissue, destruct tumor cells, and inhibit the proliferation of virus cells. B lymphocyte, as humoral immunity, is activated by an antigen and differentiated into plasma cells producing kinds of antibodies. KGM intervened the CTX‐induced immunosuppressive model, as shown as Figure 1. Compared with control group, CTX treatment extremely significantly reduced the proliferation of splenic lymphocytes (*p* < .01). Compared with CTX group, KGM could significantly promote the proliferation of B lymphocytes at the low dose (*p* < .01). The decreasing tendency was reversed with the addition of KGM, but beginning to decline at the high dose. The middle dose of KGM extremely significantly improved the proliferation of T lymphocytes induced by Con. A and B lymphocytes induced by LPS (*p* < .01). It was speculated that the middle dose of KGM could enhance humoral immunity and cell immunity, but the high dose of KGM inhibited the immune function of lymphocyte. Therefore, the metabolic load of the mice may be increased by the high dose of KGM, but both cellular immunity and humoral immunity of mice can be enhanced by the middle dose of KGM.

### 3.3 Effects on serum hemolysin level

**Figure 2:**
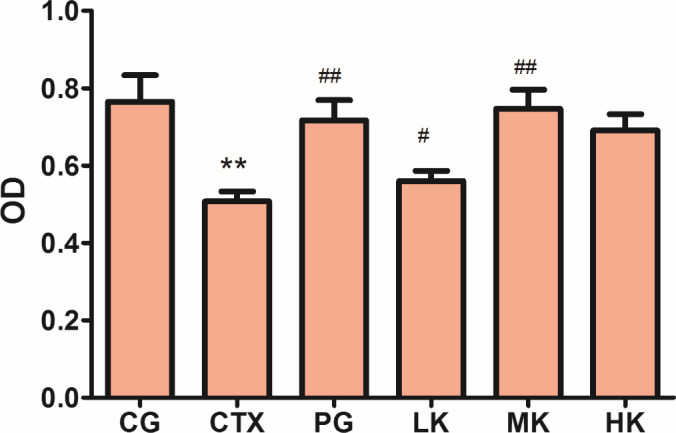
KGM demonstrated significant effects on serum hemolysin level treated by CTX. Values are expressed as means ± *SD*, *n* = 8. *p < .05, **p < .01, Compared with control group; ^#^ *p* < .05, ^##^ *p* < .01, compared with CTX group. CG, control group; CTX, CTX model group; PG, positive group; LK, low KGM; MK, middle KGM; HK, high KGM

The content of serum hemolysin is an important index of humoral immunity. Serum hemolysin which is specific antibodies is produced by organism stimulated by SRBC (Zhang & Huang, 2005). Under certain conditions, complexes formed by serum hemolysin and SRBC in vivo and in vitro, which binds to the complement will cause SRBC hemolysis, where the degree of hemolysis is related to the amount of antibody secreted (Zhao et al., 2014). The content of serum hemolysin could be further investigated the influence of KGM on the humoral immune, in this study. As shown in Figure 2, CTX treatment extremely significantly reduced the level of serum hemolysin (*p* < .01). Compared with CTX group, the middle dose of KGM could extremely significantly improve the production of serum hemolysin (*p* < .01), but not significantly compared with PG (*p* > .05). It was demonstrated that KGM could improve the level of serum hemolysin to increase the number of antibodies, which indicated that KGM could enhance and regulate humoral immune activity via serum hemolysin.

### 3.4 Effects on the NK cell lethality

**Figure 3:**
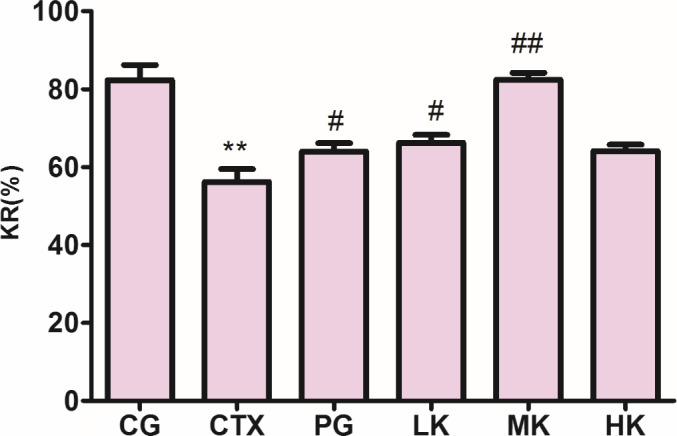
KGM demonstrated significant effects on the NK cell lethality treated by CTX. Values are expressed as means ± *SD*, *n* = 8. *p < .05, **p < .01, compared with control group; ^#^ *p* < .05, ^##^ *p* < .01, compared with CTX group. CG, control group; CTX, CTX model group; PG, positive group; LK, low KGM; MK, middle KGM; HK, high KGM

NK cells existing in the organism are the ideal effector cells, which could dissolve and clear some tumor cells and virus‐infected cells without antigenic stimulation and antibody presence (Surayot & You, 2017; Yu et al., 2014). Tumor cells, virus‐infected cells, and some abnormal self‐tissue fineness are the targets of NK cells which are closely related to antitumor, anti‐infection, and immune regulation (Huyan et al., 2014). The NK cell lethality, as an important index, could judge the immunity of organism. As shown as Figure 3, CTX could very obviously reduce the capacity of NK cell lethality (*p* < .01). Before the high dose of KGM, the capacity of NK cell lethality was obviously promoted in a dose‐dependent manner, but the capacity of NK cell lethality was more significant at the middle dose of KGM (*p* < .01) than the low dose (*p* < .05). In contrast, the capacity of NK cell lethality treated by LMS in positive group was less about 29% than KGM groups. Therefore, it was proved that KGM could extremely obviously protect the NK cell lethality from decreasing at the middle dose in boosting the immunity of organism.

### 3.5 Effects on phagocytic activity of mouse peritoneal macrophages

**Figure 4:**
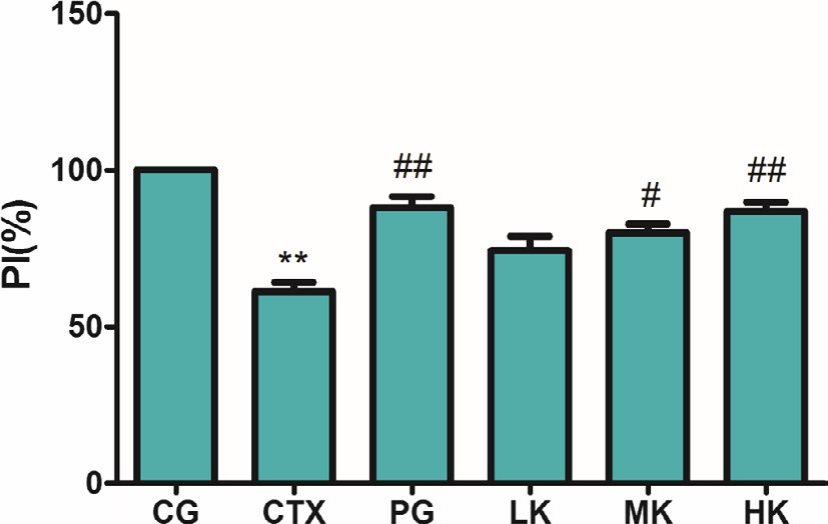
KGM demonstrated significant influences on phagocytic activity of mouse peritoneal macrophages treated by CTX. Values are expressed as means ± *SD*, *n* = 8. *p < .05, **p < .01, compared with control group; ^#^ *p* < .05, ^##^ *p* < .01, compared with CTX group. CG, control group; CTX, CTX model group; PG, positive group; LK, low KGM; MK, middle KGM; HK, high KGM

Macrophages, as an important immune cell, derived from monocytes, which are very important effector cells in nonspecific immune response play a very important role in regulating innate and acquired immunity (Govers et al., 2016). Macrophages recognize and phagocytize invading pathogens, and then present pathogens into the T lymphocytes to initiate an immune response (Liu et al., 2018). The phagocytic ability of macrophages which represents the activity of phagocytes is an important factor to investigate the innate immune function in defense mechanism against foreign substances and maintaining stability in vivo (Jang et al., 2014; Liu et al., 2018). As known as from Figure 4, it was found that the phagocytic activity of peritoneal macrophages was extremely significantly reduced in CTX treatment group (*p* < .01). Compared with CTX group, the phagocytic activities of peritoneal macrophages were significantly influenced by KGM‐intervention group (*p* < .05) where they were dose‐dependent. Especially in the high dose group, the phagocytic activities of peritoneal macrophages were increased by 21.28%. In summary, KGM could enhance the immunocompetence of CTX‐induced suppression mice but not the large dose. It was demonstrated that KGM could enhance the phagocytic ability of macrophages to activate the innate immune response against foreign substances and maintained stability in vivo.

### 3.6 Effects on the levels of cytokines

**Figure 5:**
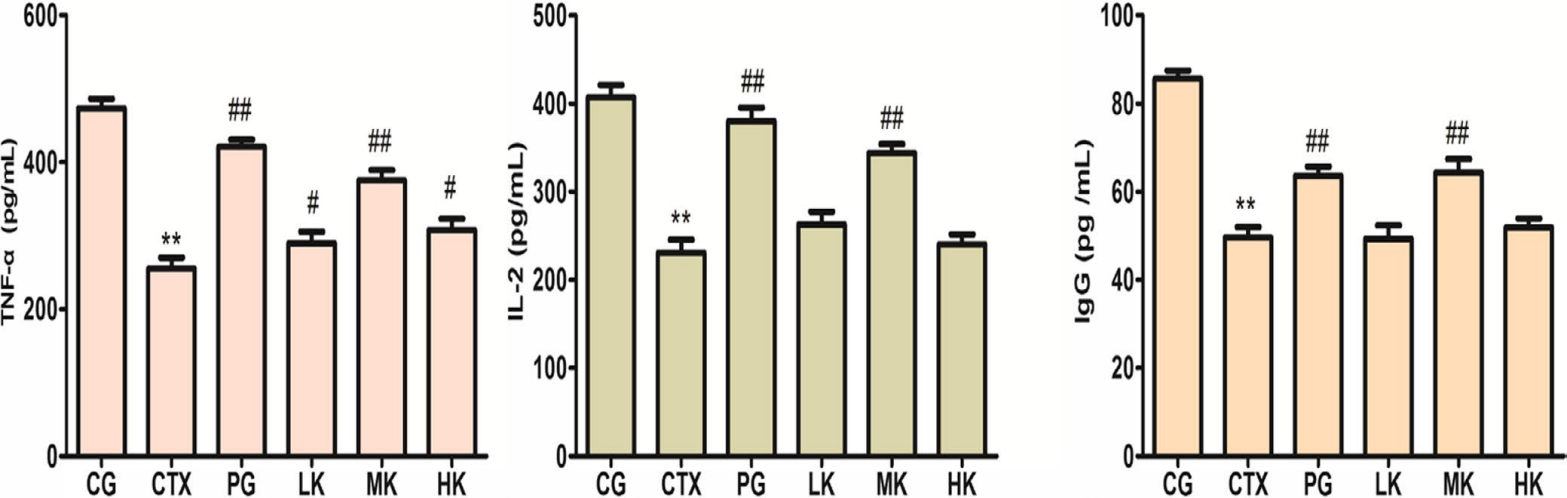
KGM demonstrated significant influences on levels of cytokines treated by CTX. a, Effect of KGM on TNF‐α production in serum; b, Effect of KGM on IL‐2 production in serum; c, Effect of KGM on IgG production in serum. Values are expressed as means ± *SD*, *n* = 8. *p < .05, **p < .01, compared with control group; ^#^ *p* < .05, ^##^ *p* < .01, compared with CTX group. CG, control group; CTX, CTX model group; PG, positive group; LK, low KGM; MK, middle KGM; HK, high KGM

The immune response associated with various levels of cytokines is a multistep process. TNF‐α, secreted by mononuclear‐macrophage, which is produced by almost all immune cells and many other cells could regulate inflammation to eliminate the tumor plays a critical role in the immune system (Yu et al., 2016). As shown as Figure 5a, CTX treatment could express the reduction in the production of IL‐2, IgG, and TNF‐α. The production of TNF‐α was significantly improved in three intervention groups (*p* < .05), but the best at the middle dose. In Figure 5b, compared with control group, KGM could very significantly improve the production of IL‐2 at three doses (*p* < .01), but the best at the middle dose too.

IL‐2, as an important lymphocyte growth factor, whose content in serum also reflects the activity and function of lymphocytes. IL‐2 produced by T lymphocytes is responsible for the proliferation and differentiation of T lymphocytes to mediate immune response, such as improving the capacity of the NK cell lethality and stimulating macrophages (Liao et al., 2011). The same production of IL‐2 was presented between positive group and the high KGM group. Compared with model group, B lymphocyte proliferation was no significant difference (*p* > .05). It was speculated that the different immune functions appeared between IL‐2 and B lymphocyte proliferation.

IgG, as immunoglobulin and major serum albumin, was produced by B lymphocyte in the humoral immune response to protect immunity through the lymphocytes activated and differentiated into an immunoglobulin‐producing plasma cell, when IgG contacted with an antigen (Verduyn et al., 2019). As known as Figure 5c, the production of IgG was improved at three doses, but compared with model group, it was extremely obviously increased at the middle dose (*p* < .01).

As shown as Figure 5(a‐c), after KGM treatment, the levels of TNF‐α, IL‐2, and IgG returned to 411.12 pg/ml, 378.79 pg/ml, and 73.59 pg/ml, respectively. In brief, KGM could significantly boost the production of cytokines (TNF‐α, IL‐2, IgG) in a dose‐dependent manner, which indicated that KGM could enhance the CTX‐induced immunosuppressed mice.

## 4 CONCLUSIONS

In this study, we found that KGM could recover the immune effect on CTX‐induced immunosuppressive mice through inhibiting the attenuation of thymus and spleen, enhancing the proliferation of T/B lymphocyte, serum hemolysin level, phagocytic activity, and the capacity of the NK cell lethality, inducing cells to secrete cytokines (TNF‐α, IL‐2, and IgG). In summary, these results showed that KGM had the potential immunomodulatory activity, so it could be applied as a potent adjuvant and an antitumor agent in functional food.

## CONFLICT OF INTEREST

We certify that there is no conflict of interest with any financial organization regarding the material discussed in the manuscript.

## ETHICAL APPROVAL

This study was approved by the ethics committee at Wannan Medical College (approval number 2018‐LLSC‐001).

## Data Availability

Research data are not shared because of the nature of the research.
